# Supplementary material for: Pooled prevalence and associated factors of chronic undernutrition among under-five children in East Africa: A multilevel analysis
Source: PLoS One. 2021 Mar 25;16(3):e0248637. doi: 10.1371/journal.pone.0248637 (PMC7993805; doi:10.1371/journal.pone.0248637)
Supplement: Attachment — Submitted filename: PONE-D-20-18977_reviewer.docx [file pone.0248637.s001.docx]

Manuscript	[Click here to access/download;Manuscript;Manuscript.docx](https://www.editorialmanager.com/pone/download.aspx?id=27081783&guid=162d5dab-8e6c-429b-aa89-d805f14fee36&scheme=1)

Pooled prevalence and associated factors of chronic undernutrition among under- five children in East Africa: A multilevel analysis

Getayeneh Antehunegn Tesema^1*^, Yigizie Yeshaw^1,2^, Misganaw Gebrie Worku^3^, Zemenu Tadesse Tessema^1^, Achamyeleh Birhanu Teshale^1^

Email Address:

[GAT-getayenehantehunegn@gmail.com](mailto:GAT-getayenehantehunegn@gmail.com)

YY- [yigizieyeshaw29@gmail.com](mailto:yigizieyeshaw29@gmail.com)

MGW- [misgeb2008@gmail.com](mailto:misgeb2008@gmail.com)

ZTT- [zemenut1979@gmail.com](mailto:zemenut1979@gmail.com)

[ABT-achambir08@gmail.com](mailto:ABT-achambir08@gmail.com)

^1^Department of Epidemiology and Biostatistics, institute of public health, college of medicine and health science, University of Gondar, Ethiopia

^2^department human physiology, school of medicine, of the college of medicine and health science, University of Gondar, Ethiopia

^3^department human anatomy, school of medicine, of the college of medicine and health science, University of Gondar, Ethiopia

* Corresponding author Getayeneh Antehunegn

Email; [getayenehantehunegn@gmail.com](mailto:getayenehantehunegn@gmail.com)

## Abstract

**Background**: Childhood undernutrition continues to be the leading cause of under-five mortality and morbidity in the world, particularly in East Africa. Although there are studies on child undernutrition in different East African countries, our search of the literature revealed that there is limited evidence of a pooled analysis of these studies. Therefore, this study aimed to investigate the pooled prevalence and associated factors of chronic undernutrition (i.e. stunting) among under-five children in East Africa.

**Methods**: A pooled analysis of the Demographic and Health Surveys (DHSs) in 12 East African countries was conducted. A total weighted sample of 79744 under-five children was included in the study. Mixed-effect logistic regression analysis was used to identify significant factors associated with chronic undernutrition since the DHS data has a hierarchical structure. The intra-class correlation coefficient (ICC), Median Odds Ratio (MOR), Likelihood Ratio (LR)-test, and deviance was used for model comparison. Variables with p-value <0.2 in the bivariable mixed-effect logistic regression analysis were considered for the multivariable analysis. In the multivariable multilevel analysis model, the Adjusted Odds Ratio (AOR) with the 95% Confidence Interval (CI) of significant factors associated with chronic undernutrition were reported.

**Results**: The pooled prevalence of chronic undernutrition among under-five children in East Africa was 33.3% (95% CI: 32.9%, 35.6%) ranging from 21.9% in Kenya to 53% in Burundi. Children whose mother lived in rural area (AOR=1.11, 95% CI: 1.06, 1.16), mothers aged 25- 34 (AOR=0.83, 95% CI: 0.79, 0.86) and ≥ 35 years (AOR=0.76, 95% CI: 0.72, 0.81), born to

mother who had no formal education (AOR=1.42, 95% CI: 1.34, 1.50) and primary education (AOR=1.37, 95% CI: 1.31, 1.44), being in poor household (AOR=1.66, 95% CI: 1.58, 1.74),

and middle household (AOR=1.42, 95% CI: 1.35, 1.49), child aged 36-48 months (AOR=1.09,

95% CI: 1.04, 1.14), being male (AOR=1.19, 95% CI: 1.15, 1.23), being multiple births

(AOR=1.98, 95% CI: 1.81, 2.17), ANC visit (AOR=0.89, 95% CI: 0.86, 0.93), small size at

birth (AOR=1.35, 95% CI: 1.29, 1.40), large size at birth (AOR=0.85, 95% CI: 0.81, 0.88), 2-

4^th^ order birth (AOR=1.08, 95% CI: 1.03, 1.13), and above 4^th^ 1.27 (AOR=1.27, 95% CI: 1.19,

1.35), home delivery 1.09 (AOR=1.09, 95% CI: 1.05, 1.13), and family size >8 (AOR=0.92, 95% CI: 0.87, 0.98) were significantly associated with stunting among under-five children.

**Conclusion**: The study revealed that stunting among under-five children remains a major public health problem in East Africa. Public health interventions should target rural residents, multiple births, mothers who had a lower level of education, and home delivery to reduce child under-nutrition, since the majority of these factors can be preventable.

**Keywords**: Stunting; East Africa; DHS; Mixed effect analysis

# Background

Adequate nutrition is vital for healthy growth, and neurological and cognitive development in early childhood [1]. The first 1000 days after birth are the critical period for infant growth and development [2]. Stunting is a linear growth failure that causes both physical and cognitive delays in growth and development [3] and it is the best predictor of chronic child undernutrition and household well-being [4]. Stuntingis defined as the inability to attain potential height for age and under-five children are particularly vulnerable to malnutrition [5]. A child is considered to be stunted when their height for age is minus two standard deviations below the median population [6].

Malnutrition is the leading cause of under-five morbidity and mortality [7]. Globally, an estimated 178 million under-five children are stunted, of whom more than 90% live in Africa and Asia [8]. It affects one-third of under-five children in low and middle- income countries [9]. The prevalence of stunting among under-5 children is highest in Africa (36%), of these the largest number of stunted children is found in East Africa [10]. More than half (54%) of under-five mortality each year are due to malnutrition and its related complications [11].

It is well known that childhood malnutrition has significant consequences on child growth and development [12, 13]. It has both short and long term adverse effects [14]. The short term consequences are recurrent infections [15], increased disease severity [18] and delayed recovery from disease [16], delayed physical and mental development [17], whereas the long term consequences of stunting are poor academic performance [19], premature death

[13] and increased risk of chronic diseases such as DM, HTN and heart disease [20, 21].

In addition, malnutrition has a significant negative consequence on the future reproductive health of female children [22, 23].

Previous studies on stunting among under-five children revealed that the causes of stunting are multifactorial [24-27]. Commonly reported predictors of stunting among under-five children include residence, duration of breastfeeding, wealth status, media exposure, maternal education, husband education, antenatal care (ANC) visit during pregnancy, postnatal care (PNC), place of delivery, health care access, women decision making autonomy, childhood illness, congenital diseases, maternal BMI, birth order and family size [28-32].

According to UNICEF data, the global progress of stunting has been declined from 255 million to 159 million from 1990 to 2014 [33]. Nevertheless, today, one in four infants in East African countries is stunted [34]. Despite the Sustainable Development Goal (SDG) ambitious target to reduce stunting among children by 40% by 2025 [35, 36], the prevalence in East Africa remains unacceptably high [37, 38].

Though there are studies conducted on stunting in East African countries [28-32], these were mainly based on hospital records, and these studies are unable to capture the pooled prevalence and associated factors of stunting among under-five children in East Africa. However, the current study was based on weighted nationally representative data from East African countries. Therefore, this study aimed to investigate the pooled prevalence and associated factors of chronic undernutrition among under-five children in East Africa. The study findings could help to inform the design of evidence-based public health interventions for reducing childhood under-nutrition, and subsequently reduce child mortality, in East Africa.

# Methods

## Data source

A secondary data analysis was done based on the most recent Demographic and Health Surveys (DHS) conducted in the 12 East African countries (Burundi, Ethiopia, Comoros, Uganda, Rwanda, Tanzania, Mozambique, Madagascar, Zimbabwe, Kenya, Zambia, and Malawi).

These datasets were merged to determine the pooled prevalence and associated factors of stunting among under-five children in East Africa. The DHS is a nationally representative survey that collects data on basic health indicators like mortality, morbidity, family planning service utilization, fertility, maternal and child health. The data were derived from the *MEASURE DHS program (https://dhsprogram.com*.) The DHS has different datasets (men, women, children, birth,

and household datasets). For this study, we used the child data set (KR file). In the KR file, all children who were born in the last five years preceding the survey in the selected enumeration area were included. The DHS used a two stages stratified sampling technique to select the study participants. We pooled the DHS surveys conducted in the 12 East African countries and a total weighted sample of 79744 under-five children were included in the study.

## Variables of the study

The outcome variable was stunted (chronic undernutrition) or not stunted in children aged 6- 59 months. In DHS to assess whether a child was stunted or not, the height for age measurement status was used. Children with a height for age measurement of < -2 standard deviation from the median of the reference population were considered to be short for their age (stunted), and children with a measurement of ≥ 2 standard deviation units were considered as not stunted. The response variable for the i^th^ child is represented by a random variable Yi with two possible values coded as 1 and 0. So, the response variable of the i^th^ child Yi was measured as a dichotomous variable with possible values Yi = 1, if the child was stunted and Yi = 0 if a child was not stunted.

The independent variables were classified into four themes as community-level, maternal, household, and child-related variables. Community-level factors included were the place of residence, country, and distance to the health facility. The maternal factors included were maternal age, maternal education, marital status, maternal Body Mass Index (BMI), woman’s health care decision making autonomy, maternal occupation, place of delivery, the mode of delivery, ANC visit during pregnancy, PNC, media exposure, paternal education, and maternal height. Child-related variables included the sex of the child, age of the child, type of birth, birth order, child-size at birth, exclusive breastfeeding for 6 month, and nutritional status (wasting, and underweight). The household factors included covered by health insurance, type of toilet, source of fuel for cooking, wealth status, sex of household head, and family size.

Media exposure was calculated by aggregating three variables such as watching television, listening to the radio, and reading newspapers. Then divided as having media exposure if a mother has been exposed to at least one of the three and not if she had no exposure to any of the media sources.

## Data management and analysis

We pooled the DHS data from the 12 East African countries together after extracting the variables based on the literature. Before any statistical analysis, the data were weighted using sampling weight, primary sampling unit, and strata to restore the representativeness of the survey and to account for sampling design when calculating standard errors and reliable estimates. "Svy set" STATA command was used for analysis to take in to account the complex survey design. Cross tabulations and summary statistics were done using STATA version 14 software. The pooled prevalence of stunting with the 95% Confidence Interval (CI) was reported using a forest plot. The DHS data had a hierarchical nature, this could violate the independence of observations and equal variance assumption of the traditional logistic regression model. Hence, children are nested within a cluster and we expect that children within the same cluster are more likely to be related to each other than children in another cluster. This implies that there is a need to take into account the between cluster variability by using advanced models. Therefore, for the associated factors, we used the mixed-effect logistic regression analysis method. Model comparison and fitness were assessed based on the Intra-class Correlation Coefficient (ICC), Likelihood Ratio (LR) test, Median Odds Ratio (MOR), and deviance (- 2LLR) values since the models were nested. Accordingly, a mixed effect logistic regression model (both fixed and random effect) was the best-fitted model since it had the lowest deviance value. Variables with p-value <0.2 in the bi-variable analysis were considered in the multivariable mixed-effect logistic regression model. Adjusted Odds Ratios (AOR) with a 95% Confidence Interval (CI) and p-value ≤ 0.05 in the multivariable model were used to declare significant factors associated with stunting.

# Ethics considerations

Ethical approval and participant consent were not necessary for this particular study since the study was a secondary data analysis based on the publically available DHS data. We requested the data from the MEASURE DHS Program and permission was granted to download and use the data for this study. There are no names of individuals or household addresses in the data files.

# Results

## Characteristics of the study population

A total of 79744 under-five children were included in the study, of these 40171 (50.4%) were males. The median age of children was 31 (IQR±13.5) months. About 19.7% of the children were from Kenya and the majority (76.2%) of the children were living in rural areas. The majority of mothers had attained primary level of education (51.6%) or higher (23.1%) and 49.4% were aged 25-34 years. The majority (68.6%) of children were delivered at a health facility (Table 1).

## Prevalence of stunting among under-five children in East Africa

The pooled prevalence of stunting among under-five children in East Africa was 33.3% (95 % CI: 32.9, 35.6). The prevalence was varied across countries, it was highest in Burundi (53%) and lowest in Kenya (21.9%) (Figure 1).

Figure 1: The prevalence of stunting among under-five children in East African countries

## Associated factors of stunting among under-five children

### Random effect results

The mixed-effect logistic regression model was the best-fitted model for the data because of the smallest value of deviance (Table 2). Furthermore, the ICC value in the null model was 18.9% [95% CI: 15.3%, 23.3%], it showed that about 18.9% of the total variability of stunting among under-five children was attributed to the between cluster variability whereas the remaining 81.1% of the total variability was explained by the between-individual variation. The MOR was 1.61, and showed that if we randomly select two children from two different clusters, a child from a cluster with a high risk of stunting was 1.61 times more likely to be stunted than a child from the cluster with a lower risk of stunting. Furthermore, the likelihood ratio test was (LR test vs. Logistic model: chibar2 (01) = 6623.18, Prob >= chiba2 = <0.001) significant which informed that the mixed-effect logistic regression model (GLMM) is the better model over the basic model (Table 2).

### The fixed effect results

In the multivariable mixed-effect logistic regression analysis; residence, country, maternal age, maternal education, wealth status, child age, sex of the child, type of birth, place of delivery, ANC visit, child-size at birth, birth order and family size were significantly associated with stunting among under-five children. The odds of stunting among children living in the rural area were 1.11 (AOR=1.11, 95% CI: 1.06, 1.16) times higher compared to those children in

urban areas. Children in Burundi, Comoros, Ethiopia, Madagascar, Malawi, Mozambique, Rwanda, Tanzania, Uganda, Zambia and Zambia were 4.76 (AOR=4.76, 95% CI: 4.40, 5.14),

1.36 (AOR=1.36, 95% CI: 1.22, 1.52), 1.88 (AOR=1.88, 95% CI: 1.75, 2.02), 3.44 (AOR=3.44,

3.18, 3.72), 1.89 (AOR=1.89, 95% CI: 1.74, 2.05), 2.51 (AOR=2.51, 95% CI: 2.34, 2.70), 2.41

(AOR=2.41, 95% CI: 2.20, 2.64), 1.78 (AOR=1.78, 95% CI: 1.66, 1.91), 1.30 (AOR=1.30,

95% CI: 1.19, 1.42), 2.43 (AOR=2.43, 95% CI: 2.28, 2.59), and 1.45 (AOR=1.45, 95% CI:

1.32, 1.59) times higher odds of stunting than children in Kenya respectively. The odds of stunting for children born to mothers aged 25-34 and ≥ 35 years were decreased by 17% (AOR=0.83, 95% CI: 0.79, 0.86) and 24% (AOR=0.76, 95% CI: 0.72, 0.81) compared to children born to mothers aged 15-24 years, respectively. Children born to mothers who had no formal education and primary education had 1.42 (AOR=1.42, 95% CI: 1.34, 1.50) and 1.37 (AOR=1.37, 95% CI: 1.31, 1.44) times higher likelihood to be stunted than children born to mothers who attained secondary education and above, respectively. Children born to poor wealth households and middle wealth households had 1.66 (AOR=1.66, 95% CI: 1.58, 1.74), and 1.42 (AOR=1.42, 95% CI: 1.35, 1.49) times higher odds of stunting compared to children born to the rich wealth household.

Children aged 36-47.9 months were 1.09 times (AOR=1.09, 95% CI: 1.04, 1.14) higher odds of stunting compared to children aged < 24 months. Being male increased the odds of stunting by 19% (AOR=1.19, 95% CI: 1.15, 1.23) compared to female children. The odds of stunting among multiple births were 1.98 times (AOR=1.98, 95% CI: 1.81, 2.17) higher than singletons. The odds of stunting among children whose mother had ANC visits during pregnancy were decreased by 11% (AOR=0.89, 95% CI: 0.86, 0.93) than children whose mother didn’t have ANC during pregnancy. Children who were small size at birth were 1.35 (AOR=1.35, 95% CI: 1.29, 1.40) times more likely to be stunted compared to those children who were average size at birth whereas the odds of stunting among children who were large size at birth were decreased by 15% (AOR=0.85, 95% CI: 0.81, 0.88) compared to average size children at birth. Children who were 2-4^th^ born and above 4^th^ born had 1.08 (AOR=1.08, 95% CI: 1.03, 1.13), and 1.27 (AOR=1.27, 95% CI: 1.19, 1.35) times more likely to be stunted as compared to the first birth. The odds of stunting among children belonging to households with eight and above family members were decreased by 8% (AOR=0.92, 95% CI: 0.87, 0.98). Children born at home were 1.09 (AOR=1.09, 95% CI: 1.05, 1.13) times more likely to be stunted as compared to children born at a health facility (Table 3).

# Discussion

This study provides evidence that stunting among under-five children continues to be a major public health problem in East African countries. Our study investigated the pooled prevalence and associated factors of stunting among under-five children in East African countries to understand the overall prevalence and associated factors of stunting in East Africa as well as the inter-country distribution of stunting among under-five children across the East African countries.

In this study, 33.3% of under-five children in East African countries were stunted. This is lower than the study reported in Sub-Saharan Africa [38] but higher than a study finding in Iran [39]. This could be due to East African countries being more vulnerable to food shortages because they rely on agriculture which is highly sensitive to weather and climate conditions such as temperature, precipitation, and light and extreme events and low capacity for adaptation [40, 41]. Furthermore, stunting among under-five children is strongly associated with poverty [42, 43]. Iran is relatively wealthier and has good access to basic maternal and child health care services compared to East African countries, hence this could be the possible explanation for the decreased prevalence of stunting in Iran.

In the multivariable mixed-effect logistic regression; residence, country, maternal age, maternal education, wealth status, child age, sex of the child, type of birth, place of delivery, ANC visit, child-size at birth, birth order and family size were significantly associated with stunting. In this study, under-five children in a rural area had higher odds of stunting than urban children. It was consistent with study findings [44, 45], this could be due to the reason that urban residents have good access to education, improved access to water and sanitation facilities that contributed to having good child nutrition. Besides, in urban areas maternal health care services such as ANC, health facility delivery, and PNC visit are available, that could raise community awareness to provide quality complementary feeding and uses child immunization services, this could contribute for the lower risk of child stunting in urban areas [46].

The odds of stunting for children born to mothers aged 25-34 and ≥ 35 years were lower than children born to mothers aged 15-24 years. It was in line with the study reported in Nigeria [47]. The possible explanation could be due to the reason that teenagers who gave birth are less likely to use health services like vaccination services, and have poor health-seeking behavior relative to mature mothers. Children born to mothers with a lower level of education were more likely to be stunted compared to children born to mothers who attained a secondary and higher level of education. It is consistent with the study findings in Nairobi [48] and Bolivia [49]. It could be because educated mothers have good knowledge about child health and basic health care services, and an enhanced capacity to recognize childhood illness and seek treatment for their children [50]. Besides, educated breastfeed exclusively for 6 months and provide recommended complementary feeding compared to uneducated mothers [51].

In our study wealth status is one of the most important predictors of stunting. Children in the poor and middle household's wealth were more likely to be stunted than children in the richest household wealth. This was consistent with studies in Indonesia [52], Bangladesh [53] and Angola [54], and stunting is considered to be a sensitive indicator of food insecurity

and poverty [55]. Children who live in poor households take inadequate food, poor access to safe water, and hygiene [56]. Consequently, they are at higher risk of childhood infectious diseases such as acute respiratory diseases, diarrheal diseases, and intestinal parasites, all of which contribute to chronic undernutrition [57]. Children in the age group of 36-48 months were more likely to be stunted compared to children aged less than 24 months. It is consistent with a study reported in Nigeria [47], this could be due to the reason that as children ages increase they had environmental exposure that could increase their risk of infections like tonsillopharyngitis, diarrheal diseases, pneumonia and CROUP [58, 59]. The risk of malnutrition is highest mainly after the initiation of complementary feeding that has been directly linked with poor complementary feeding and breastfeeding weaning practices of mothers, these together with high rates of infectious diseases could be the possible reason for the increased risk of stunting in older children [60, 61].

Males were more likely to be stunted than females. This is supported by previous study findings reported in SSA [62], Pakistan [63], and Brazil [64]. This could be due to the slower lung maturation among males compared to females that predispose male children to repeated respiratory infections such as pneumonia, bronchiolitis, otitis media, and hyperactive airway disease which could contribute to the increased risk of stunting among males [65]. Children born as multiple births are at higher risk of being stunted compared to singletons. It is consistent with studies reported in India [66] and Nigeria [67], and the increased risk of stunting is likely explained by the fact that multiple births are more likely to be born prematurely ,be of lower birth weight, and experience increased competition for nutrition [68]. In our study, health facility delivery and ANC visit are the significant predictors of reduced risk of child chronic undernutrition. This finding is supported by studies reported in Indonesia [69] and Nepal [70], this could be due to the reason that ANC visit is an entry point for the other maternal health services, and births from mother who had an ANC visit and health facility delivery are aware of the danger signs of childhood illness, using childhood immunization services and appropriate childhood feeding practice [71].

Children who were small size at birth were more likely to be stunted whereas large-sized babies at birth had lower odds of being stunted as compared to children who were average size at birth. This was consistent with studies [72, 73], and could be due to the increased vulnerability of small size babies to infections mainly diarrheal and lower respiratory infections such as pneumonia, and otitis media, and increased risk of complications including sleep apnea, anemia, chronic lung disorders, fatigue and loss of appetite compared to children with normal birth weights [74, 75]. In our study, birth order and, family size were significant predictors of stunting among under-five children. Children of 2 and above birth order were more likely to be stunted than children of first birth order, which was in line with previous study findings [76, 77]. This might be due to large-sized families being unable to satisfy the dietary and other healthcare-related service needs of their children, as well as due to maternal nutrition depletion [78].

The strength of this study was that it was based on a weighted large, nationally representative data set with adequate statistical power to detect the true association of factors with stunting among under-five children. Besides, the study was conducted using an advanced model to take into account the clustering effect (mixed-effect logistic regression) to get reliable standard error and estimate. However, the study finding is interpreted in light of limitations. First, as with other cross-sectional studies, the temporal relationship can't be established. Second, the DHS didn’t incorporate information about health care availability and accessibility like distance to the health facility, medical-related factors, and the quality of maternal health services provided which might influence child nutrition status. Also, since data was collected from self-report from respondents there may be a possibility of social desirability and recall bias.

# Conclusion

The pooled prevalence of stunting among under-five children in East Africa was 33.3% ranging from 21.9% in Kenya to 53% in Burundi. Chronic child undernutrition continues to be a major public health problem in East Africa. In this study; residence, country, maternal age, maternal education, wealth status, child age, sex of the child, type of birth, place of delivery, ANC visit, child-size at birth, birth order, and family size were significantly associated with stunting among under-five children. Therefore, to improve child nutritional status governmental and non- governmental organizations should design public health interventions targeting rural residents, and the poorest households. Furthermore enhancing health facility delivery, ANC visits, and maternal health education is vital for reducing child chronic undernutrition.

# Abbreviations

ANC; Antenatal Care, AOR; Adjusted Odds Ratio; CI; Confidence Interval, DHS; Demographic Health Survey, GLMM; Generalized Linear Mixed Models, ICC; Intra-class Correlation Coefficient; LLR; log-likelihood Ratio, LR; Likelihood Ratio, MOR; Median Odds Ratio; SSA; Sub-Saharan Africa, WHO; World Health Organization

# Declarations

## Ethical approval and consent to participate

Since the study was a secondary data analysis of publically available survey data from the MEASURE DHS program, ethical approval and participant consent were not necessary for this particular study. We requested DHS Program and permission was granted to download and use the data for this study from [http://www.dhsprogram.com.](http://www.dhsprogram.com/) There are no names of individuals or household addresses in the data files.

## Consent for publication

Not applicable since the study was a secondary data analysis

## Availability of data and materials

Data is available online and can be accessed from [www.measuredhs.com.](http://www.measuredhs.com/)

## Competing Interests

Authors declare that they have no conflict of interest

## Funding

No funding was obtained for this study.

## Authors contribution

GAT, ABT, YY, MGW and ZTT conceived the study. GAT, ABT, YY, MGW and ZTT analyzed the data, drafted the manuscript, and reviewed the article. GAT, ABT, YY, MGW and ZTT extensively reviewed the article. All authors read and approved the final manuscript.

## Acknowledgments

We greatly acknowledge MEASURE DHS for granting access to the East African DHS data sets.

# References

1. Martorell R: **The nature of child malnutrition and its long-term implications**. *Food and nutrition Bulletin* 1999, **20**(3):288-292.
2. Smith LC, Haddad L: **Reducing child undernutrition: past drivers and priorities for the post-MDG era**. *World Development* 2015, **68**:180-204.
3. Kandala N-B, Madungu TP, Emina JB, Nzita KP, Cappuccio FP: **Malnutrition among children under the age of five in the Democratic Republic of Congo (DRC): does geographic location matter?** *BMC public health* 2011, **11**(1):261.
4. Som S, Pal M, Bharati P: **Role of individual and household level factors on stunting: A comparative study in three Indian states**. *Annals of Human Biology* 2007, **34**(6):632-646.
5. Walker SP, Gaskin PS, Powell CA, Bennett FI: **The effects of birth weight and postnatal linear growth retardation on body mass index, fatness and fat distribution in mid and late childhood**. *Public Health Nutrition* 2002, **5**(3):391-396.
6. Leroy JL, Ruel M, Habicht J-P, Frongillo EA: **Using height-for-age differences (HAD) instead of height- for-age z-scores (HAZ) for the meaningful measurement of population-level catch-up in linear growth in children less than 5 years of age**. *BMC pediatrics* 2015, **15**(1):145.
7. Bryce J, Boschi-Pinto C, Shibuya K, Black RE, Group WCHER: **WHO estimates of the causes of death in children**. *The Lancet* 2005, **365**(9465):1147-1152.
8. Prendergast AJ, Humphrey JH: **The stunting syndrome in developing countries**. *Paediatrics and international child health* 2014, **34**(4):250-265.
9. Sunguya BF, Ong KI, Dhakal S, Mlunde LB, Shibanuma A, Yasuoka J, Jimba M: **Strong nutrition governance is a key to addressing nutrition transition in low and middle-income countries: review of countries’ nutrition policies**. *Nutrition journal* 2014, **13**(1):65.
10. Bloss E, Wainaina F, Bailey RC: **Prevalence and predictors of underweight, stunting, and wasting among children aged 5 and under in western Kenya**. *Journal of tropical pediatrics* 2004, **50**(5):260- 270.
11. Schofield C, Ashworth A: **Why have mortality rates for severe malnutrition remained so high?**

*Bulletin of the World Health Organization* 1996, **74**(2):223.

12. De Onis M, Branca F: **Childhood stunting: a global perspective**. *Maternal & child nutrition* 2016, **12**:12- 26.
13. Dewey KG, Begum K: **Long‐term consequences of stunting in early life**. *Maternal & child nutrition*

2011, **7**:5-18.

14. Reinhardt K, Fanzo J: **Addressing chronic malnutrition through multi-sectoral, sustainable approaches: a review of the causes and consequences**. *Frontiers in nutrition* 2014, **1**:13.
15. Guerrant RL, DeBoer MD, Moore SR, Scharf RJ, Lima AA: **The impoverished gut—a triple burden of diarrhoea, stunting and chronic disease**. *Nature reviews Gastroenterology & hepatology* 2013, **10**(4):220.
16. Hendrikse W, Reilly J, Weaver L: **Malnutrition in a children's hospital**. *Clinical Nutrition* 1997, **16**(1):13- 18.
17. Branca F, Ferrari M: **Impact of micronutrient deficiencies on growth: the stunting syndrome**. *Annals of Nutrition and Metabolism* 2002, **46**(Suppl. 1):8-17.
18. Lelijveld N, Seal A, Wells JC, Kirkby J, Opondo C, Chimwezi E, Bunn J, Bandsma R, Heyderman RS, Nyirenda MJ: **Chronic disease outcomes after severe acute malnutrition in Malawian children (ChroSAM): a cohort study**. *The Lancet Global Health* 2016, **4**(9):e654-e662.
19. Haile D, Nigatu D, Gashaw K, Demelash H: **Height for age z score and cognitive function are associated with Academic performance among school children aged 8–11 years old**. *Archives of Public Health* 2016, **74**(1):17.
20. Uauy R, Kain J, Mericq V, Rojas J, Corvalán C: **Nutrition, child growth, and chronic disease prevention**. *Annals of medicine* 2008, **40**(1):11-20.
21. Sawaya AL, Sesso R, Florêncio TMdMT, Fernandes MT, Martins PA: **Association between chronic undernutrition and hypertension**. *Maternal & child nutrition* 2005, **1**(3):155-163.
22. Konje JC, Ladipo OA: **Nutrition and obstructed labor**. *The American journal of clinical nutrition*

2000, **72**(1):291S-297S.

23. Kruger H: **Maternal anthropometry and pregnancy outcomes: a proposal for the monitoring of pregnancy weight gain in outpatient clinics in South Africa**. *Curationis* 2005, **28**(4):40-49.
24. Tiwari R, Ausman LM, Agho KE: **Determinants of stunting and severe stunting among under-fives: evidence from the 2011 Nepal Demographic and Health Survey**. *BMC pediatrics* 2014, **14**(1):239.
25. Teshome B, Kogi-Makau W, Getahun Z, Taye G: **Magnitude and determinants of stunting in children underfive years of age in food surplus region of Ethiopia: the case of west gojam zone**. *Ethiopian Journal of Health Development* 2009, **23**(2).
26. Chirande L, Charwe D, Mbwana H, Victor R, Kimboka S, Issaka AI, Baines SK, Dibley MJ, Agho KE: **Determinants of stunting and severe stunting among under-fives in Tanzania: evidence from the 2010 cross-sectional household survey**. *BMC pediatrics* 2015, **15**(1):165.
27. Shrimpton R, Kachondham Y: **Analysing the causes of child stunting in DPRK**. *New York: UNICEF*

2003.

28. Torlesse H, Cronin AA, Sebayang SK, Nandy R: **Determinants of stunting in Indonesian children: evidence from a cross-sectional survey indicate a prominent role for the water, sanitation and hygiene sector in stunting reduction**. *BMC public health* 2016, **16**(1):669.
29. Deshmukh PR, Sinha N, Dongre AR: **Social determinants of stunting in rural area of Wardha, Central India**. *medical journal armed forces india* 2013, **69**(3):213-217.
30. Aguayo VM, Nair R, Badgaiyan N, Krishna V: **Determinants of stunting and poor linear growth in children under 2 years of age in India: An in‐depth analysis of Maharashtra's comprehensive nutrition survey**. *Maternal & child nutrition* 2016, **12**:121-140.
31. Keino S, Plasqui G, Ettyang G, van den Borne B: **Determinants of stunting and overweight among young children and adolescents in sub-Saharan Africa**. *Food and nutrition bulletin* 2014, **35**(2):167- 178.
32. Vella V, Tomkins A, BORGESI A, Migliori GB, Oryem VY: **Determinants of stunting and recovery from stunting in northwest Uganda**. *International journal of epidemiology* 1994, **23**(4):782-786.
33. Shekar M, Kakietek J, Dayton Eberwein J, Akuoku JK, Pereira A: **Reaching the Global Target for Stunting**. In*.*, edn.; 2017.
34. Akombi BJ, Agho KE, Hall JJ, Wali N, Renzaho A, Merom D: **Stunting, wasting and underweight in sub-Saharan Africa: a systematic review**. *International journal of environmental research and public health* 2017, **14**(8):863.
35. Haddad LJ, Hawkes C, Achadi E, Ahuja A, Ag Bendech M, Bhatia K, Bhutta Z, Blossner M, Borghi E, Eriksen K: **Global Nutrition Report 2015: Actions and accountability to advance nutrition and sustainable development**: Intl Food Policy Res Inst; 2015.
36. Fan S, Polman P: **An ambitious development goal: Ending hunger and undernutrition by 2025**: Intl Food Policy Res Inst; 2014.
37. De Onis M, Blössner M, Borghi E: **Prevalence and trends of stunting among pre-school children, 1990–2020**. *Public health nutrition* 2012, **15**(1):142-148.
38. Akombi BJ, Agho KE, Merom D, Renzaho AM, Hall JJ: **Child malnutrition in sub-Saharan Africa: A meta-analysis of demographic and health surveys (2006-2016)**. *PloS one* 2017, **12**(5).
39. Mansori K, Shadmani FK, Mirzaei H, Azad RV, Khateri S, Hanis SM, Moradi Y: **Prevalence of stunting in Iranian children under five years of age: Systematic review and meta-analysis**. *Medical journal of the Islamic Republic of Iran* 2018, **32**:103.
40. Kadi K, Hame A, Njau L, Mwikya J, Kamga A: **The state of climate information services for agriculture and food security in East African countries**. 2011.
41. Ferguson EL, Gibson RS, Thompson LU, Ounpuu S: **Dietary calcium, phytate, and zinc intakes and the calcium, phytate, and zinc molar ratios of the diets of a selected group of East African children**. *The American journal of clinical nutrition* 1989, **50**(6):1450-1456.
42. Hassanzadeh J, Mohammadbeigi A, Eshrati B, Rezaianzadeh A, Rajaeefard A: **Determinants of inequity in health care services utilization in markazi province of iran**. *Iranian Red Crescent Medical Journal* 2013, **15**(5):363.
43. Ghodsi D, Omidvar N, Eini-Zinab H, Rashidian A, Raghfar H: **Impact of the national food supplementary program for children on household food security and maternal weight status in Iran**. *International journal of preventive medicine* 2016, **7**.
44. Melaku YA, Zello GA, Gill TK, Adams RJ, Shi Z: **Prevalence and factors associated with stunting and thinness among adolescent students in Northern Ethiopia: a comparison to World Health Organization standards**. *Archives of Public Health* 2015, **73**(1):44.
45. Menon P, Ruel MT, Morris SS: **Socio-economic differentials in child stunting are consistently larger in urban than in rural areas**. *Food and Nutrition Bulletin* 2000, **21**(3):282-289.
46. Smith LC, Ruel MT, Ndiaye A: **Why is child malnutrition lower in urban than in rural areas? Evidence from 36 developing countries**. *World development* 2005, **33**(8):1285-1305.
47. Akombi BJ, Agho KE, Hall JJ, Merom D, Astell-Burt T, Renzaho AM: **Stunting and severe stunting among children under-5 years in Nigeria: A multilevel analysis**. *BMC pediatrics* 2017, **17**(1):15.
48. Taffa N, Chepngeno G: **Determinants of health care seeking for childhood illnesses in Nairobi slums**.

*Tropical Medicine & International Health* 2005, **10**(3):240-245.

49. Frost MB, Forste R, Haas DW: **Maternal education and child nutritional status in Bolivia: finding the links**. *Social science & medicine* 2005, **60**(2):395-407.
50. Basu AM, Stephenson R: **Low levels of maternal education and the proximate determinants of childhood mortality: a little learning is not a dangerous thing**. *Social science & medicine* 2005, **60**(9):2011-2023.
51. Khattak AM, Gul S, Muntaha ST: **Evaluation of nutritional knowledge of mothers about their children**. *Gomal Journal of Medical Sciences* 2007, **5**(1).
52. Gross R, Schultink W, Sastroamidjojo S: **Stunting as an indicator for health and wealth: an Indonesian application**. *Nutrition research* 1996, **16**(11-12):1829-1837.
53. Hong R, Banta JE, Betancourt JA: **Relationship between household wealth inequality and chronic childhood under-nutrition in Bangladesh**. *International journal for equity in health* 2006, **5**(1):15.
54. Kennedy G, Nantel G, Brouwer ID, Kok FJ: **Does living in an urban environment confer advantages for childhood nutritional status? Analysis of disparities in nutritional status by wealth and residence in Angola, Central African Republic and Senegal**. *Public Health Nutrition* 2006, **9**(2):187-193.
55. Reinhard I, Wijeratne K: **The use of stunting and wasting as indicators for food insecurity and poverty**: Poverty Impact Monitoring Unit; 2000.
56. Chilton M, Chyatte M, Breaux J: **The negative effects of poverty & food insecurity on child development**. *Indian Journal of Medical Research* 2007, **126**(4):262.
57. Kikafunda JK, Walker AF, Collett D, Tumwine JK: **Risk factors for early childhood malnutrition in Uganda**. *Pediatrics* 1998, **102**(4):e45-e45.
58. Riley ID: **Acute respiratory infections**. In: *Handbook of Nutrition and Immunity.* edn.: Springer; 2004: 265-286.
59. Kjellén M: **Health and environment**: Sida; 2001.
60. Organization WH: **Essential nutrition actions: improving maternal, newborn, infant and young child health and nutrition**. 2013.
61. Underwood BA, Hofvander Y: **Appropriate timing for complementary feeding of the breast‐fed infant: a review**. *Acta Pædiatrica* 1982, **71**:1-32.
62. Wamani H, Åstrøm AN, Peterson S, Tumwine JK, Tylleskär T: **Boys are more stunted than girls in sub-Saharan Africa: a meta-analysis of 16 demographic and health surveys**. *BMC pediatrics* 2007, **7**(1):17.
63. Khuwaja S, Selwyn BJ, Shah SM: **Prevalence and correlates of stunting among primary school children in rural areas of southern Pakistan**. *Journal of tropical pediatrics* 2005, **51**(2):72-77.
64. Hoffman DJ, Sawaya AL, Coward WA, Wright A, Martins PA, de Nascimento C, Tucker KL, Roberts SB: **Energy expenditure of stunted and nonstunted boys and girls living in the shantytowns of Sao Paulo, Brazil**. *The American journal of clinical nutrition* 2000, **72**(4):1025-1031.
65. Adamson I, Bakowska J, McMillan E, King G: **Accelerated fetal lung maturation by estrogen is associated with an epithelial-fibroblast interaction**. *In Vitro Cellular &Developmental Biology* 1990, **26**(8):784-790.
66. Fenske N, Burns J, Hothorn T, Rehfuess EA: **Understanding child stunting in India: a comprehensive analysis of socio-economic, nutritional and environmental determinants using additive quantile regression**. *PloS one* 2013, **8**(11).
67. Adekanmbi VT, Kayode GA, Uthman OA: **Individual and contextual factors associated with childhood stunting in Nigeria: a multilevel analysis**. *Maternal & child nutrition* 2013, **9**(2):244-259.
68. Medhin G, Hanlon C, Dewey M, Alem A, Tesfaye F, Worku B, Tomlinson M, Prince M: **Prevalence and predictors of undernutrition among infants aged six and twelve months in Butajira, Ethiopia: the P- MaMiE Birth Cohort**. *BMC public health* 2010, **10**(1):27.
69. Agho KE, Inder KJ, Bowe SJ, Jacobs J, Dibley MJ: **Prevalence and risk factors for stunting and severe stunting among under-fives in North Maluku province of Indonesia**. *BMC pediatrics* 2009, **9**(1):64.
70. Paudel R, Pradhan B, Wagle R, Pahari D, Onta S: **Risk factors for stunting among children: a community based case control study in Nepal**. *Kathmandu University Medical Journal* 2012, **10**(3):18- 24.
71. Pembe AB, Carlstedt A, Urassa DP, Lindmark G, Nyström L, Darj E: **Quality of antenatal care in rural Tanzania: counselling on pregnancy danger signs**. *BMC pregnancy and childbirth* 2010, **10**(1):35.
72. Aryastami NK, Shankar A, Kusumawardani N, Besral B, Jahari AB, Achadi E: **Low birth weight was the most dominant predictor associated with stunting among children aged 12–23 months in Indonesia**. *BMC Nutrition* 2017, **3**(1):16.
73. Addo OY, Stein AD, Fall CH, Gigante DP, Guntupalli AM, Horta BL, Kuzawa CW, Lee N, Norris SA, Prabhakaran P: **Maternal height and child growth patterns**. *The Journal of pediatrics* 2013, **163**(2):549-554. e541.
74. Rahman MS, Howlader T, Masud MS, Rahman ML: **Association of low-birth weight with malnutrition in children under five years in Bangladesh: Do mother’s education, socio-economic status, and birth interval matter?** *PloS one* 2016, **11**(6).
75. Mukhopadhyay K, Mahajan R, Louis D, Narang A: **Longitudinal growth of very low birth weight neonates during first year of life and risk factors for malnutrition in a developing country**. *Acta Paediatrica* 2013, **102**(3):278-281.
76. Moges B, Feleke A, Meseret S, Doyore F: **Magnitude of Stunting and Associated Factors Among 6-59 Months Old Children in Hossana Town, Southern Ethiopia**. *Journal of Clinical Research & Bioethics* 2015, **6**(1):1.
77. Rahman A, Chowdhury S: **Determinants of chronic malnutrition among preschool children in Bangladesh**. *Journal of biosocial science* 2007, **39**(2):161-173.
78. Allen LH: **Causes of nutrition-related public health problems of preschool children: available diet**. In*.*: LWW; 2006.

Table 1: Maternal, child, household and community level characteristics of the study population in East Africa (N=79744)

| **Variables** | **Weighted frequency (%)** |
| --- | --- |
| **Community level variables** |  |
| **Country** |  |
| Burundi | 5580 (7.0) |
| Comoros | 2221 (2.8) |
| Ethiopia | 8455 (10.6) |
| Kenya | 15705 (19.7) |
| Madagascar | 4522 (5.7) |
| Malawi | 4635 (5.8) |
| Mozambique | 8818 (11.1) |
| Rwanda | 3258 (4.1) |
| Tanzania | 7782 (9.8) |
| Uganda | 3881 (4.9) |
| Zambia | 10259 (12.8) |
| Zimbabwe | 4624 (5.8) |
| **Residence** |  |
| Rural | 60751 (76.2) |
| Urban | 18993 (23.8) |
| **Distance to health facility** |  |
| Not a big problem | 47147 (59.1) |
| A big problem | 32597 (40.9) |
| **Maternal related characteristics** |  |
| **Mothers age** |  |
| 15-24 | 21556 (27.0) |
| 25-34 | 39398 (49.4) |
| 35-49 | 18790 (23.6) |
| **Maternal education status** |  |
| No | 20178 (25.3) |
| Primary | 41170 (51.6) |
| Secondary and higher | 18396 (23.1) |
| **Marital status** |  |
| Never married | 59331 (74.4) |
| Married/living together | 11337 (14.2) |
| Divorced/widowed/separated | 9076 (11.4) |
| **Working status** |  |
| No | 27941 (35.0) |
| Yes | 51803 (65.0) |
| **Maternal BMI** |  |
| Underweight | 15834 (19.9) |
| Normal | 49750 (62.4) |
| Overweight | 14160 (17.7) |

### Woman’s health care decision making autonomy

Respondent alone	14389 (18.0)

Jointly with husband/partner	31519 (39.5)

| Husband/partner only | 33836 (42.5) |
| --- | --- |
| **Media exposure** |  |
| No | 26221 (32.9) |
| Yes | 53523 (67.1) |
| **Husband education status** |  |
| No | 14503 (18.2) |
| Primary | 32181 (40.4) |
| Secondary and above | 33060 (41.4) |
| **Maternal height** |  |
| Short | 9371 (11.8) |
| Normal | 70373 (88.2) |
| **Place of delivery** |  |
| Home | 25055 (31.4) |
| Health facility | 54689 (68.6) |
| **Mode of delivery** |  |
| Vaginal | 75240 (94.3) |
| Caesarean | 4504 (5.7) |
| **ANC visit during pregnancy** |  |
| No | 28566 (35.8) |
| Yes | 51177 (64.2) |
| **PNC visit (n=41430)** |  |
| No | 23295 (56.2) |
| Yes | 18135 (43.8) |
| **Household level characteristics** |  |
| **Type of toilet facility** |  |
| Improved | 29686 (37.2) |
| Not improved | 50058 (62.8) |
| **Source of fuel** |  |
| Modern fuel | 5137 (6.4) |
| Traditional fuel | 74607 (93.6) |
| **Covered by health insurance** |  |
| No | 63723 (79.9) |
| Yes | 16021 (20.1) |
| **Wealth status** |  |
| Poor | 36630 (45.9) |
| Middle | 15670 (19.7) |
| Rich | 27444 (34.4) |
| **Sex of household head** |  |
| Male | 61510 (77.1) |
| Female | 18234 (22.9) |
| **Family size** |  |
| 1-4 | 23288 (29.2) |
| 5-8 | 44878 (56.3) |
| ≥ 9 | 11578 (14.5) |
| **Child characteristics** |  |
| **Sex of child** |  |
| Male | 40171 (50.4) |
| Female | 39573 (49.6) |

| **Age of child (in months)** |  |
| --- | --- |
| < 24 | 28195 (35.3) |
| 24-35.9 | 17524 (22.0) |
| 36-47.9 | 17295 (21.7) |
| 48-60 | 16730 (21.0) |
| **Type of birth** |  |
| Single | 77558 (97.3) |
| Twin | 2186 (2.7) |
| **Birth order** |  |
| First | 17399 (21.8) |
| 2-4 | 38958 (48.9) |
| ≥ 4 | 23387 (29.3) |
| **Exclusively breast feed for 6 months** |  |
| No | 69273 (86.7) |
| Yes | 10471 (13.2) |
| **Child size at birth** |  |
| Small | 20114 (25.2) |
| Average | 36513 (45.8) |
| Large | 23117 (29.0) |
| **Wasting status** |  |
| Normal | 76044 (95.4) |
| Wasted | 3700 (4.6) |
| **Underweight status** |  |
| Normal | 15767 (19.8) |
| Underweight | 63977 (80.2) |

Table 2: Model comparison and model fitness

| Parameter | Standard logistic regression model Null model Mixed-effect logistic regression model |
| --- | --- |
| LLR | -47182.02 -47117.89 |
| Deviance | 94364.04 94235.6 |
| AIC | 94436.04 94309.77 |
| BIC | 94770.29 94653.35 |
| ICC | 18.9% (95% CI: 15.3%,  23.3%) |
| MOR | 1.61 (1.57, 1.65) |
| LR-test | LR test vs. logistic model: chibar2(01) = 245.15 Prob >= chibar2 = 0.001 |

Table3: The bi-variable and multivariable mixed-effect logistic regression analysis of stunting among under 5 children in East Africa

| **Variable** | **Stunted** | **Not stunted** | **COR with 95% CI** | **AOR with 95% CI** |
| --- | --- | --- | --- | --- |
| **Residence** |  |  |  |  |
| Urban | 14535 | 4458 | 1 | 1 |
| Rural | 38691 | 22060 | 1.79 (1.72, 1.86) | 1.11 (1.06, 1.16)^*^ |
| **Country** |  |  |  |  |
| Kenya | 12262 | 3443 | 1 | 1 |
| Comoros | 1597 | 3076 | 1.15 (1.03, 1.27) | 1.36 (1.22, 1.52)^*^ |
| Ethiopia | 5379 | 3076 | 1.78 (1.68, 1.89) | 1.88 (1.75, 2.20)^*^ |
| Burundi | 2620 | 2960 | 3.60 (3.37, 3.85) | 4.76 (4.40, 5.14)^*^ |
| Madagascar | 2302 | 2220 | 2.95 (2.74, 3.17) | 3.44 (3.18, 3.72)^*^ |
| Malawi | 3175 | 1460 | 1.48 (1.37, 1.59) | 1.89 (1.74, 2.05)^*^ |
| Mozambique | 5361 | 3457 | 1.88 (1.77, 2.00) | 2.51 (2.34, 2.70)^*^ |
| Rwanda | 2113 | 1145 | 1.77 (1.63, 1.93) | 2.41 (2.20, 2.64)^*^ |
| Tanzania | 5410 | 2372 | 1.42 (1.33, 1.51) | 1.78 (1.66, 1.91)^*^ |
| Uganda | 2943 | 939 | 1.09 (1.01, 1.19) | 1.30 (1.19, 1.42)^*^ |
| Zambia | 6475 | 3784 | 1.93 (1.82, 2.04) | 2.43 (2.28, 2.59)^*^ |
| Zimbabwe | 3588 | 1036 | 0.89 (0.82, 0.97) | 1.45 (1.32, 1.59)^*^ |
| **Maternal age** |  |  |  |  |
| 15-24 | 14032 | 7523 | 1 | 1 |
| 25-34 | 26785 | 12613 | 0.91 (0.88, 0.95) | 0.83 (0.79, 0.86)^*^ |
| ≥ 35 | 12409 | 6381 | 0.98 (0.94, 1.02) | 0.76 (0.72, 0.81)^*^ |
| **Maternal education** |  |  |  |  |
| No | 11791 | 8387 | 2.40 (2.29, 2.51) | 1.42 (1.34, 1.50)^**^ |
| Primary | 27003 | 14167 | 1.90 (1.83, 1.98) | 1.37 (1.31, 1.44)^*^ |
| Secondary and above | 14431 | 3964 | 1 | 1 |
| **Wealth status** |  |  |  |  |
| Poor | 22149 | 14481 | 2.04 (1.97, 2.11) | 1.66 (1.58, 1.74)^**^ |
| Middle | 10256 | 5413 | 1.67 (1.60, 1.75) | 1.42 (1.35, 1.49)^*^ |

| Rich | 20820 | 6624 | 1 | 1 |
| --- | --- | --- | --- | --- |
| **Media exposure** |  |  |  |  |
| Yes | 37494 | 16028 | 1 | 1 |
| No | 17732 | 10489 | 1.52 (1.47, 1.56) | 1.04 (0.99, 1.08) |
| **Women health care decision making autonomy** | | | | |
| Respondent alone | 9640 | 4749 | 1 | 1 |
| Jointly with husband/ partner | 20759 | 10760 | 1.06 (1.02, 1.11) | 0.99 (0.94, 1.03) |
| Husband/partner only | 22823 | 11008 | 1.03 (0.99, 1.08) | 1.04 (0.99, 1.09) |
| **Distance to health facility** |  |  |  |  |
| Not a big problem | 32729 | 14417 | 1 | 1 |
| A big problem | 20496 | 12101 | 1.33 (1.29, 1.37) | 1.02 (0.98, 1.06) |
| **Childs age in months** |  |  |  |  |
| <24 | 19061 | 9134 | 1 | 1 |
| 24-35.9 | 11810 | 5714 | 1.03 (0.99, 1.08) | 1.01 (0.97, 1.06) |
| 36-47.9 | 11284 | 6011 | 1.12 (1.07, 1.16) | 1.09 (1.04, 1.14)^*^ |
| 48-60 | 11070 | 5660 | 1.08 (1.03, 1.12) | 1.02 (0.97, 1.07) |
| **Sex of child** |  |  |  |  |
| Male | 26127 | 14043 | 1.15 (1.12, 1.19) | 1.19 (1.15, 1.23)^*^ |
| Female | 27098 | 12475 | 1 | 1 |
| **Type of birth** |  |  |  |  |
| Single | 52082 | 25475 | 1 | 1 |
| Multiple | 1143 | 1044 | 2.04 (1.87, 2.22) | 1.98 (1.81, 2.17)^**^ |
| **Place of delivery** |  |  |  |  |
| Health facility | 37519 | 17168 | 1 | 1 |
| Home | 15707 | 9348 | 1.29 (1.25, 1.34) | 1.09 (1.05, 1.13)^*^ |
| **ANC visit during pregnancy** |  |  |  |  |
| No | 17874 | 10692 | 1 | 1 |
| Yes | 35352 | 15826 | 0.75 (0.73, 0.78) | 0.89 (0.86, 0.93)^*^ |
| **Exclusively breast feed for 6 months** | | | | |
| No | 6941 | 3530 | 1 | 1 |
| Yes | 46285 | 22988 | 0.92 (0.88, 0.96) | 0.95 (0.89, 1.01) |

| **Child size at birth** |  | | | |
| --- | --- | --- | --- | --- |
| Average | 24347 | 12166 | 1 | 1 |
| Small | 13104 | 7010 | 1.12 (1.08, 1.16) | 1.35 (1.29, 1.40)^**^ |
| Large | 15775 | 7342 | 0.89 (0.86, 0.92) | 0.85 (0.81, 0.88)^**^ |
| **Birth order** |  |  |  |  |
| First | 11987 | 5412 | 1 | 1 |
| 2-4 | 26557 | 12401 | 1.06 (1.02, 1.10) | 1.08 (1.03, 1.13)^**^ |
| ≥ 5 | 14682 | 8706 | 1.33 (1.28, 1.39) | 1.27 (1.19, 1.35)^*^ |
| **Family size** |  |  |  |  |
| 1-4 | 15871 | 7416 | 1 | 1 |
| 5-8 | 29520 | 15358 | 1.15 (1.11, 1.19) | 1.03 (0.99, 1.07) |
| >8 | 7835 | 3744 | 1.05 (1.01, 1.10) | 0.92 (0.87, 0.98)^*^ |

Figure	[Click here to access/download;Figure;Figure 1.tif](https://www.editorialmanager.com/pone/download.aspx?id=26851149&guid=0532e124-603e-4682-81a6-b85e91fa9874&scheme=1)

For consistency please report the Burundi prevalence to one decimal point i.e. 53.0
