# Supplementary material for: Pooled prevalence and associated factors of chronic undernutrition among under-five children in East Africa: A multilevel analysis
Source: PLoS One. 2021 Mar 25;16(3):e0248637. doi: 10.1371/journal.pone.0248637 (PMC7993805; doi:10.1371/journal.pone.0248637)
Supplement: Attachment — Submitted filename: Point by point response.docx [file pone.0248637.s002.docx]

**PLOS ONE**

**Point by point response for editors/reviewers comments**

**Manuscript title:** Pooled prevalence and associated factors of chronic undernutrition among under-five children in East Africa: A multilevel analysis

**Manuscript ID: PONE-D-20-18977**

**Dear editor/reviewer.**

Dear all,

We would like to thank you for these constructive, building, and improvable comments on this manuscript that would improve the substance and content of the manuscript. We considered each comment and reviewers on the manuscript thoroughly. Our point-by-point responses for each comment and question are described in detail on the following pages.

**Response to reviewers comments**

**Reviewer#1**

1. Abstract

Under the results; the third sentence should read "Children whose mothers" not mother.

**Authors’ response**: Thank you for the comments. We accept and modified it**. (See Abstract section, line 41, page 2)**

2. Key words

The authors can consider to add stunting, pooled prevalence, associated factors, under-five children, East-Africa, DHS . Remove "mixed effect analysis".

**Authors’ response**: Thank you for the concerns. We removed it in the revised manuscript. (See Abstract section, line 57, line 3)

3. Background

first paragraph sentence 7 - A child is considered stunted... The authors should remove "as"

second paragraph this sentence is missing something and should be rephrased "Malnutrition is the leading cause of under-five morbidity and mortality". Is it under-five 'children'?

**Authors’ response**: Thank you for the comments. We accepted and modified it. (See Background section, page 4)

4. Results

This sentence "The median age of children was 31 (IQR±13.5) months" should either report median (IQR) or mean±SD. It cant be IQR and SD.

**Authors’ response**: Thank you for the comments. We reported the median as a measure of central tendency and Inter-quartile Rage (IQR) as a measure of dispersion since the variable was not normally distributed (skewed) with Shapiro Wilks test p-value<0.05.

**Reviewer #2**

**1.** In general, this is a well conducted study although the writing is awkward in places with some grammatical errors. A lack of line numbering made it difficult to highlight these issues so I have edited the paper and I attach a copy of this paper with changes marked. Further suggestions for improvements are listed below.

**Authors’ response**: Thank you reviewer for the detailed comments and changes you made for the betterment of the paper. Sorry for the missing line number, and now, we insert the line number and address the comments you raised. (See the revised manuscript)

2. Abstract

The results section of the abstract contains a single, very long sentence which contains all of the factors significantly associated with undernutrition. I suggest breaking this into two sentences which first identifies those factors associated with an increased risk of stunting and then a second sentence which identifies those factors which were associated with a decreased risk of stunting.

**Authors’ response**: Thank you for the comments. We accept the comments and write accordingly. (See Abstract section, line 39 -52, page 2-3)

3. Background

Is the statistic of 1 in 4 infants in East African countries being stunted actually for infants (i.e. children under 12 months of age) or for children under-five years, which is the target group for this study?

**Authors’ response**: Thank you for the comments. We rewrote it as our target population is under-five children. (See the revised manuscript)

4. Methods

The explanatory variable ‘exclusive breastfeeding’ needs to be more clearly defined. Is this a continuous variable which indicates the duration of EBF or is it a binary variable which indicates whether a child was EBF to 6 months of age? The results in table 4 suggest it is the latter.

**Authors’ response**: Thank you for the comments. We consider the duration of EBF as a binary outcome by categorizing those children who breastfeed for a minimum of 6 months exclusively as yes and for those children who feed less than months as no. (See the revised manuscript)

5. Results

5. Under the data management section you say that the pooled prevalence of stunting with the 95% Confidence Interval (CI) was reported using a forest plot. However, while you report the pooled prevalence in the results section you have not included the forest plot.

**Authors’ response**: Thank you for the comments. We planned to present using a forest plot but we prefer to present it in a bar graph as this study was not a metanalysis. As you know while we have done, in the forest plot several columns were presented like weight but this may not be important to present it. So, we presented the pooled prevalence in the bar graph. (See Figure 1)

6. When reporting the findings from the multivariable mixed-effect logistic regression analysis you describe each variable individually and you move between variables that are associated with an increased risk of stunting and those with a decreased risk of stunting. I think it would be clearer if you were to first present those variables associated with a higher risk of stunting and then in a new paragraph identify those that are associated with a lower risk of stunting

**Authors’ response**: Thank you for the comments. We accept the comments and report the findings from factors that increase the odds of stunting to factors that decrease the odds of stunting. (See the Result section, line 195 – 228, page 9-11)

7. The sentence that compares the odds of stunting for each country compared to Kenya is very long and repeats results that are easily discernible from the table. I suggest simplifying this finding and only provide the results for the countries with the lowest and highest odds ratios e.g. “Compared with Kenya which had the lowest prevalence of stunting, all of the other countries had a significantly higher odds of stunting ranging from an odds of 1.30 (1.19, 1.42) for Uganda to 4.76 (4.40, 5.14) for Burundi.”

**Authors’ response**: Thank you for the comments. We revised it. (See Result section, line 202-203, page 10)

8. PNC is described in the methods section as an explanatory variable. While it is reported in table 1 there is no further reference to PNC. This variable isn’t included in Table 3 or reported in the results or discussion in terms of its association with stunting. You should either remove all reference to PNC or redo the analysis with this variable in the model. Or does table 3 only contain those variables which had a p-value above 0.2 in the bivariable analysis? This is not clear if this is the case.

**Authors’ response**: Thank you for the concern. We consider PNC in the method and result section. But, in the multivariable multilevel analysis, we did not use PNC as it has a p-value>0.2 in the bivariable analysis. As we stated in the method section we consider variables with a p-value in the bivariable multilevel analysis were included in the multivariable multilevel analysis, that is why we did not include PNC in the final model.

9. Discussion

For clarity, I suggest discussing all of the factors associated with an increased risk of stunting and THEN discussing those factors associated with a decreased risk. You move between factors associated with increased and decreased risk and then back to increased risk.

**Authors’ response**: Thank you for the suggestions. We accept it and rewrote it. (See discussion section)

10. References

A number of the references are very dated e.g. 1, 11, 16, 32, 41, 52, 57, 61, 65. Are they seminal papers or can they be replaced with more recent references?

Reference 7 The last ‘author’ appears to be a group/organisation but it is unclear which group. When entering the details of a group put a comma after the last word of the name to denote that this is a group. E.g.

WHO Child Health Epidemiology Reference Group,

Otherwise ENDNOTE treats the last word as the family name and initialises all of the other words e.g. Group,WCHERG

Reference 33 Some details appear to be missing. Is this a book or a chapter of a book?

Reference 35 Please do not abbreviate the institution which has published the report. Where was this report published?

Reference 59 Reference details are incomplete.

**Authors’ response:** Thank you for the comments. We modified all the suggested references. (See the revised manuscript)
