# Supplementary material for: Pooled prevalence and associated factors of chronic undernutrition among under-five children in East Africa: A multilevel analysis
Source: PLoS One. 2021 Mar 25;16(3):e0248637. doi: 10.1371/journal.pone.0248637 (PMC7993805; doi:10.1371/journal.pone.0248637)
Supplement: Attachment — Submitted filename: Point by point response.docx [file pone.0248637.s003.docx]

**Point by point response for editors/reviewers comments**

**PLOS ONE Journal**

**Manuscript title:** Pooled prevalence and associated factors of chronic undernutrition among under-five children in East Africa: A multilevel analysis

**Manuscript ID:**  PONE-D-20-18977R1

**Dear editor/reviewer.**

Dear all,

We would like to thank you for these constructive, building, and improvable comments on this manuscript that would improve the substance and content of the manuscript. We considered each comment and clarification questions of editors and reviewers on the manuscript thoroughly. Our point-by-point responses for each comment and question are described in detail on the following pages.

**Response to Editors**

1. Please review your reference list to ensure that it is complete and correct. If you have cited papers that have been retracted, please include the rationale for doing so in the manuscript text, or remove these references and replace them with relevant current references. Any changes to the reference list should be mentioned in the rebuttal letter that accompanies your revised manuscript. If you need to cite a retracted article, indicate the article’s retracted status in the References list and also include a citation and full reference for the retraction notice.

**Authors’ response**: Thank you for the comments. We have checked the references and it is correctly presented.

**Response to reviewers**

***Minor issues to be addressed***

1. Line 49 Being small size at birth should be included with the factors that were associated with increased odds of stunting, not those associated with a decreased odd as the AOR is greater than 1 (AOR=1.35, 95% CI: 1.29, 1.40).

Authors’ response: Thank you for the comments. We included with the factors that were associated with increased odds of stunting. (See Abstract section line 48, page 2)

2. 2. Line 55 How are ‘multiple births’ a modifiable factor unless a woman is having fertility treatment? The conclusions in your abstract should be consistent with the conclusions in your main paper on page 15

**Authors’ response**: Thank you for the comments. We rewrote it. (See Abstract section, line 53-57, page 3)

3. Line 60 you have misquoted reference 3. It is the first 1000 days ‘post conception’ NOT ‘after birth’

**Authors’ response**: Thank you for the comments. We rewrote it. (see Background section, line 61, page 4)

4. Line 304-305 The way in which you have worded this sentence makes it sound as though ‘health facility delivery and ANC visit’ are associated with an increased risk of chronic malnutrition and NOT that they are protective of chronic malnutrition. I suggest rewording this sentence

‘In our study, health facility delivery and ANC visit were protective of child chronic malnutrition.’

**Authors’ response**: Thank you for the suggestions. We accept it and revised the sentence. (See Discussion section, line 302-303, page 14)

5. Table 3 please include a footnote explaining the meaning of the asterisks. Also I recommend that you included a footnote to explain that the table includes only those variables for which the bi-variable analysis had a p <0.20, otherwise readers may wonder why some of the variables listed in Table 1 are not included in Table 2, (as I was).

**Authors’ response**: Thank you for the comments. We included the points you raised. **(See Table 3)**

**Grammatical errors**

6. line 34 should read ‘Variables’ (plural)

7. line 37 should read ‘were reported for significant factors’

8. Line 45 2nd -4th birth order

9. line 72 the word half is a collective noun and is treated as a singular. Therefore this should read ‘More than half …… is due ….’

10. line 84 the word ‘to’ is not needed, should read ‘… children include residence…..’

11. Line 89 should read ‘ has declined from..’

12. Line 139 I suggest replacing the word ‘divided’ with ‘categorised’.

13. Line 149 should read ‘presented in a bar graph’.

14. Line 195 Given that the next paragraph describes the multivariable mixed-effect logistic regression analysis, shouldn’t this subheading read ‘The mixed effect results’?

15. Lines 260 to 262 should read ‘Besides, educated women are more likely to exclusively breastfeed for….”

16. Line 266 suggest rewording this sentence ‘Children who live in poor households typically have poor access to adequate food, safe water, and sanitation.’

**Authors’ response**: We thank you a lot for your extensive effort to improve our work. We accept all the abovementioned comments and revised the manuscript. (See the revised manuscript)
